# A Mobile Virtual Butler to Bridge the Gap between Users and Ambient Assisted Living: A Smart Home Case Study

**DOI:** 10.3390/s140814302

**Published:** 2014-08-06

**Authors:** Nuno Costa, Patricio Domingues, Florentino Fdez-Riverola, António Pereira

**Affiliations:** 1 School of Technology and Management, Computer Science and Communications Research Centre, Polytechnic Institute of Leiria, Leiria 2411-901, Portugal; E-Mails: nuno.costa@ipleiria.pt (N.C.); patricio.domingues@ipleiria.pt (P.D.); 2 Higher Technical School of Computer Engineering, University of Vigo, Polytechnic Building, Campus Universitario As Lagoas s/n, 32004 Ourense, Spain; 3 INOV INESC INOVAÇÃO Instituto de Novas Tecnologias–Delegação de Leiria, Leiria 2411-901, Portugal; E-Mail: apereira@ipleiria.pt

**Keywords:** ambient intelligence, smart environments, smart objects, ubiquitous computing

## Abstract

Ambient Intelligence promises to transform current spaces into electronic environments that are responsive, assistive and sensitive to human presence. Those electronic environments will be fully populated with dozens, hundreds or even thousands of connected devices that share information and thus become intelligent. That massive wave of electronic devices will also invade everyday objects, turning them into smart entities, keeping their native features and characteristics while seamlessly promoting them to a new class of thinking and reasoning everyday objects. Although there are strong expectations that most of the users' needs can be fulfilled without their intervention, there are still situations where interaction is required. This paper presents work being done in the field of human-computer interaction, focusing on smart home environments, while being a part of a larger project called Aging Inside a Smart Home. This initiative arose as a way to deal with a large scourge of our country, where lots of elderly persons live alone in their homes, often with limited or no physical mobility. The project relies on the mobile agent computing paradigm in order to create a Virtual Butler that provides the interface between the elderly and the smart home infrastructure. The Virtual Butler is receptive to user questions, answering them according to the context and knowledge of the AISH. It is also capable of interacting with the user whenever it senses that something has gone wrong, notifying next of kin and/or medical services, *etc.* The Virtual Butler is aware of the user location and moves to the computing device which is closest to the user, in order to be always present. Its avatar can also run in handheld devices keeping its main functionality in order to track user when s/he goes out. According to the evaluation carried out, the Virtual Butler is assessed as a very interesting and loved digital friend, filling the gap between the user and the smart home. The evaluation also showed that the Virtual Butler concept can be easily ported to other types of possible smart and assistive environments like airports, hospitals, shopping malls, offices, *etc.*

## 1. Introduction

The so called Ambient Intelligence (AmI) promises a world where everyday devices can interact with each other in a supposedly smart manner to enhance usability, comfort and security. Although an important goal of AmI is to promote automation, interaction with users is still needed as the increasing number of applications for mobile and wearable devices has shown. For instance, some home lighting and climate control equipment is controlled through the manufacturer software applications, requiring the user to be knowledgeable with smart mobile devices (smartphones or tablets) and APPs. This contributes to the digital divide, where citizens with no or little exposure to modern digital devices have realize a significant effort to overcome the digital exclusion.

At the same time, as life conditions, medicine and health care improve, human beings are living longer. In fact, in a large number of countries, the average life expectancy has now surpassed 70 years, with some countries having already reached over 80 years [1]. However, although the quality of life has also improved along with the life expectancy, the senior population is still prone to have limitations regarding mobility, agility and sensorial acuity imposed by age. Additionally, it is not uncommon for a senior to live alone, making him/her more dependent on help when an abnormal situation, like a fall, occurs. For the perceived quality of life of a senior, it is important that s/he remains independent, living at the household as long as possible. Although the wealthiest citizens can hire someone to help them out such as servant personnel, the vast majority cannot afford such specialized help. In fact, many struggle with high monthly medical bills. Furthermore, senior citizens constitute a significant percentage of those excluded from the digital world (especially the low-income ones) who can seldom afford to interact with digital devices, let alone receive training. The Ambient Assisted Living (AAL) research area aims to enhance the quality of life, mainly for people who face some level of disabilities either due to age or to any other circumstance of life. The goal is to provide appropriate technological solutions to mitigate or even overcome some of the constraints felt by citizens, often living alone, with some kind of physical limitation.

With this aim in mind, over the last years, we have been developing the long-term Aging Inside a Smart Home (AISH) project in order to transform regular homes into smart homes, instrumented with sensors, actuators, wearable and mobile computing and reasoning capabilities. The architecture of the AISH project is based on an innovative fully-decoupled, rule and plug-in-based publish/subscribe core, where addressing and transport layers are abstracted through software plug-ins and application-level communication protocol is abstracted through text rules. This approach eases the device integration, promotes the interoperability among services, applications and devices and keeps the system running, even when new services or devices are being integrated.

In a highly computing populated environment, where all the devices are hidden from user eyes, new user interaction approaches are required with preference for natural ways like voice or gestures. However, resorting to such natural interaction requires a more task/objective oriented approach, departing from the current traditional human-computer interface based on menus, toolbars and alike for setting and configuring properties and actions. Indeed, interaction needs to be more human-like and coarse grained, focused on the wanted results and leaving the low level steps to the intelligent system. To this end, we have developed the Virtual Butler project, presented in this paper, in order to enhance the usability of AISH assisting technologies through a simple and affordable voice interface, using vocal commands that are correctly interpreted and executed. Reciprocally, Virtual Butler can also interact with its users through voice synthesis, either to directly respond to user's commands (“Turn TV on”) or to interact with the user, for example, to check if the user is all right. Besides being voice aware, Virtual Butler is also location aware and relies on mobile agents to virtually follow the inhabitant inside and outside home. Virtual Butler also integrates optional visual interaction through common LCD screens attached to simple and cost-effective System on a Chip (SoC) such as the Raspberry Pi. This way, computer-centered interfaces such as web interfaces and touch screens are no longer needed (nor suitable for regular usage of AISH), avoiding the trap of the digital divide. Furthermore, communicating with explicit voice commands eases the filtering out of other conversations, thus lowering the intrusion level of the Virtual Butler into the household. Indeed, respecting privacy is a mandatory issue for AAL [2]. The whole project (AISH and Virtual Butler) was evaluated in a case study using a real sensor rich environment, involving an 82 year old female in her own home with very promising results. The main contributions of this paper are as follows:

- We designed and implemented a new human-machine interaction metaphor in the Ambient Assisted Living context (the Virtual Butler) which is different and feature richer when compared with similar approaches. To the best of our knowledge, this is the first virtual butler which is interactive, receptive and location aware.
- The usage of both mobile and static agents to accomplish Virtual Butler location awareness as far as inhabitants are concerned. This allows Virtual Butler to always stay at the inhabitants' side and even travel with the inhabitant via a smartphone.
- The evaluation of the system in a real world scenario and the sharing of the learnt lessons. We had the opportunity to assess our system in an 82 year old female's home and we are sharing the real evaluation to the research community.

While this section has introduced and motivated the work, the remainder of this paper is organized as follows: Section 2 focuses on related work. Section 3 describes the architecture, while Section 4 presents the current prototype. Section 5 presents and discusses main results. Finally, Section 6 concludes the paper and presents avenues for future work.

## 2. Related Work

In this section we review related work focusing on the four main areas that bear importance to the current project: AAL, mobile software agents, voice processing with performance constrained devices and level of acceptance of senior citizens to communication devices and digital avatars.

### 2.1. Ambient Assisted Living

Ambient Assisted Living has become a research hot topic [3–13]. This is mostly due to the steep increase in life expectancy that has occurred in many countries over the last decades. Another major contributing factor lies in the mobile revolution, with ubiquitous mobile devices boosting not only processing power, but also an impressive array of sensors and communication capabilities. AAL is a subset of the much wider Ambient Intelligence (AiM) field, described by certain authors as the third wave in computing [14,15]. Our research team has been working in the area of Ambient Assisted Living on the long-term project AISH. AISH comprises multiple sub-projects, all focusing on the elderly population who have low to medium incomes and who live in rural environments in Portugal. The targeted elderly typically lives alone and frequently presents some kind of physical impairment that limits their mobility [16,17]. Examples of AISH-developed projects range from a fall detection and alert system [18–20] to interfaces aimed at breaking the isolation of the elderly population by simplifying the use of desktop and mobile applications [17]. The Virtual Butler project fits in the category of simplified interfaces, resorting to speech recognition to receive commands and voice synthesis to convey information. The emphasis in Virtual Butler is to facilitate the task of interacting with a smart home for elderly users. For this purpose, the Virtual Butler project focuses heavily, but not exclusively, on voice-based interfaces.

### 2.2. Mobile Agents

Basically, a mobile agent (MA) is an application which is able to execute relatively autonomous actions and that can migrate to the network of accessible agent servers when executing a given task. The servers provide assistance for operations such as communication, migration and access to specific services and resources like databases [21]. Major advantages of mobile agents are their mobility and autonomy, while their dependence on specific and tailored execution platforms is a weakness that hinders their portability. Mobile agents have regained a second life with the advance of powerful and connected mobile devices, as well as the ubiquity of the internet. In fact, some authors believe that mobile agent software is the Holy Grail to completely reveal the promising future of AmI environments [22].

Numerous agent platforms have been developed since the mid-90s [23], a trend partially attributed to the emergence of the Java programming language and of the Java Virtual Machine (JVM) that provided the technology for code and state portability, remote execution and code migration [24]. Examples include Aglet [25,26], Voyager, Grasshoper [27], Concordia [28], SPRINGS [29] and JADE [30–32], to name just a few. For a more comprehensive review, see the survey regarding mobile agent platforms by Nikolai and Madey [33].

JADE (short for Java Agent DEvelopment framework) is a widely used platform for mobile agents. It adheres to the FIPA standards and implements a rich set of features at the level of communication supporting several communication protocols, mobility and security, to name just a few. Contrary to other platforms, it has a long history and, most importantly, continues to be actively developed. As importantly, the platform is available under the LGPL version 2 open source license. Therefore, as it fulfills most of our requirements, the JADE platform represents a natural choice to support the mobile features of our Virtual Butler. In Section 4, we deepen the technological features of the JADE Platform that we deem important for the Virtual Butler.

### 2.3. Speech Processing and Speech Synthesis

The need for voice usage in the interaction between the smart home and its inhabitants is almost mandatory when dealing with users with little or no experience in interacting with computer-based devices as occurs with the population targeted by AISH [34,35]. In this context, Vacheret *et al.* focused on sound processing in Ambient Assisted Living [2]. Their main goal was to detect and process not only voices, but also sounds such as steps, a spinning washing machine, dishes, *etc.* Their hardware setup relies on a microphone per room of the house, seamlessly attached to the ceiling to perform sound and automatic speech recognition. Their results obtained from experiments in an AAL-ready model flat shows that sound and especially speech recognition becomes challenging in noisy environments, either due to neighborhood sources of noises (the flat used in the experiments is located near a hospital heliport) or to in-house noises like a working radio or a TV set with a medium to high volume of sound. As reported by the authors, this is mostly due to the fact that the microphones are fixed, possibly meters away from the speaking source, thus making them prone to capturing external sounds and noises. These reported difficulties for quality capture of voice when dealing with fixed microphones were the main motivation for resorting to mobile sound capture devices for the Virtual Butler project. Although this approach may seem cumbersome for the user, and indeed this was reported in our experiment, it allows for good sound capture quality and voice recognition. We also believe that the fast evolution of mobile devices will soon reduce or eliminate some of these concerns. In fact, devices such as Google Glass and Android-based smart watches are already good examples of the future.

Regarding privacy, an interesting outcome from a questionnaire performed by Vacher *et al.* points out that sounds and speech analysis are accepted as long as no recording is performed. Moreover, the questionnaire confirmed that users are totally against the usage of video for AAL [2]. Ziefle *et al.* [36] also points out that camera-based systems, followed by positioning systems, are the most disliked technology for home‐monitoring. The authors also report that microphones are the less disliked monitoring devices. In accordance with these results, Virtual Butler uses microphones for inhabitant voice capturing, thus eliminating outright the possibility of using any type of video.

The usage of voice-controlled software in mobile devices (smartphones) has brought speech and command recognition into the mainstream. Indeed, Apple's SIRI for iPhone has brought lot of media exposure and public acknowledgment of both the utilities and limitations of speech recognition in the context of so-called question-answering personal assistants [37]. This trend is being followed by Google Now [38] (Android 4.1 and above) and the still beta Cortana for Microsoft operating systems. Although SIRI currently resorts to cloud computing, thus requiring an internet connection, the Android OS supports offline speech recognition through the Google ASR service, at least in recent devices that have enough processing power [39]. This significantly eases speech recognition, a feature which is quintessential to the main core of our approach. Additionally, a growing number of smartphones uses two microphones for sound capture. This allows for noise cancellation, thus increasing the quality of the captured input sound, yielding better speech recognition. Offline speech recognition is also available through the open source CMU Sphinx—Speech Recognition Toolkit [40]. In the Virtual Butler context, we use both CMU Sphinx and Google ASR service. However, while Google ASR service is an out-of-the-box ready-to-use solution, CMU Sphinx requires low-level configuration and tuning, especially for languages other than English. Our usage of CMU Sphinx for Virtual Butler did not yield satisfactory results. To overcome this issue, we then resorted to the Google ASR service for inhabitant voice capture, which yielded much better results.

### 2.4. Level of Acceptance of Senior Citizens to Devices and Digital Avatars

The success of an AAL system is dependent of the level of changes required by the system for the targeted population. Ideally, an AAL system should require no changes, adapting itself to the targeted users and environments. In reality, and regarding AAL for senior citizens, users are often required to interact with devices. This is the case for Virtual Butler, where the elderly user needs to carry and maintain a smartphone or sound capture device and to interact with a (digital) avatar. Hwangbo *et al.* [41] report that the elderly population only makes up 11% of smartphone users. The authors attribute the low percentage usage to the difficulty to deal with interfaces with touch screens, especially when written input is required. Another contributing factor is the lack of tactile feedback. Regarding the Virtual Butler project, an important issue was the non-adaption to the smartphone, seen as a fragile and complex device. For instance, as we report later on, the elderly individual forgot to recharge the battery of the device.

Using an avatar to interact in an AmI is not a novel thing. Ortiz *et al.* [42] performed an experimental assessment of the effects of resorting to an avatar in natural interaction with 15 elderly users. Similarly to the Virtual Butler, the avatar was displayed through a flat screen. The authors reported that subjects not only followed instructions much better when interacting with the avatar, but were also able to recognize emotions in the facial expressions of the avatar. Moreover, the experience of having an avatar display emotions (smile, sadness, *etc.*) was described as a pleasant one. Our experiments with Virtual Butler have also shown that the avatar is well tolerated. However, messages through voice synthesis need to be preceded by a discrete and non-obtrusive sound to prepare the elderly for a vocal communication, in order to avoid catching the elderly by surprise.

The use of (new) metaphors for human-machine interaction in the AAL context is still a recent research topic. The *invisible computer* of Mark Weiser's dream is about to become a reality. However, we believe that autonomous computing in the future will still need to have a human-like interface, mostly based on natural language, in order to be accepted by the targeted users. The *invisible computer* will have a name and could be materialized (e.g., a robot) or virtual (e.g., a virtual pet) probably with good shape and appearance. Our research group argues that the virtual approach is the right direction due to simplicity (lack of hardware, devices drivers, battery constraints, number of points of failures, *etc.*). We have been researching how to create a preliminary proof of concept in order to evaluate it in a real world scenario. The decision to adopt a virtual butler as a metaphor for human-machine interaction came from the intrinsic aims, features and characteristics of real human butlers to help home inhabitants.

## 3. Architecture

The use of static and mobile software agents in AmI is not a new research topic. Several researchers have adopted this software paradigm in order to assist users in the context of ambient intelligence through the development of decentralized and distributed architectures [43–48]. However, these projects employ agents mainly to provide a convenient user interface to control the environment. Unlike these projects, Virtual Butler resorts to the “follow me” approach of mobile agents. For this purpose, our approach is receptive to user commands by voice, informing and alerting users by voice synthesis. It can also collect state and emotions in order to enrich the facts database, even when the users are outside their instrumented smart home.

### 3.1. The AISH Project

Virtual Butler is a component of the long-term AISH project that intents to implement the natural language interface of the system. AISH appeared as a solution to deal with a large scourge of our country (and other Western countries), where many elderly persons live alone in their homes, often with limited or no physical mobility. The main aim of AISH is to turn a traditional home into a smart home that takes care of its inhabitants by using sensors, actuators, wearable and mobile computing devices and reasoning capabilities. Hence it creates an ambient assisted living. Conceptually, AISH is composed of four main layers: (i) physical layer, (ii) integration layer, (iii) services layer and (iv) application layer.

The physical layer represents all the hardware needed to sense and actuate in the house, such as sensors, actuators or device controllers and gateways. The integration layer combines and promotes the peer communication and interoperability in an easy way, by employing a plug-in and rule-based approach where plug-ins abstract similar transport communication protocols, while rules abstract application-level communication protocols. The third layer comprises: (i) a knowledge base which stores all home actions and events, (ii) a classification and predicting decision module to dictate context-aware decisions and (iii) mash-up sensors that enable the creation of virtual sensors by coupling two or more physical sensors (e.g., a virtual fire sensor comprised of two real sensors: a temperature coupled to a smoke real sensor). Finally, the fourth layer provides application services such as alarming, remote/web administration, graphical administration console, *etc.*

**Figure 1.**
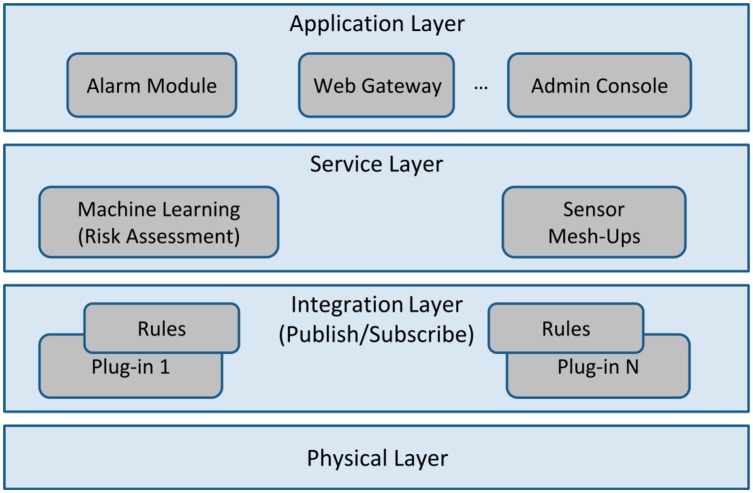
The AISH system architecture.

After finishing and evaluating the first AISH prototype, we realized that while the administration graphical console is functional and intuitive, it is not tailored for the elderly, especially people that have had few or no contact at all with computers and similar devices. Therefore, for the targeted users, a natural language interface must be used instead, while the computer savvy-oriented administration tools are restricted to be used solely by technical personnel. Hence, Virtual Butler aims to provide for an alternative to the existing administration graphical console in order to make the interface user-friendly for the targeted population. A high level overview of the architecture of the AISH project is shown in Figure 1.

### 3.2. The Virtual Butler

As reported earlier, the first version of the AISH prototype provided a graphical administration console as the main interface. In fact, the main goal of that prototype was to assess the behavior of the AISH system as a whole, with the system-human interface demoted to a secondary role, mostly oriented for computer savvy users. After successfully concluding the evaluation of the maiden prototype, we routed our efforts to provide a seamless interaction between the AISH and its users, *i.e.*, the inhabitants of the smart home. For this purpose, we quickly determined that a voice-based interface is the right path to follow, since spoken language is from an early age the natural and most used interface for human beings. Therefore, our efforts for the second prototype of AISH focused on closing the gap between the targeted users and the interface of the system, providing for a speech-based interface. Meanwhile, the computer-based interface was slightly improved, but maintaining its focus on computer savvy users. Regarding main functionalities, we target the following ones:

- Be location aware by using the knowledge of the user's whereabouts when s/he is inside the house.
- Be able to work for all supported types of interactions between human-machine.
- Be able to follow the user, that is, the interface should be available even when the user is outside the AISH house.

The first requirement demands that the interface adapt itself according to the location of the user, being location-aware. For instance, if the user is in the kitchen, the interface—waiting for user voice commands and speaking to the user—must be kitchen-focused. Whenever the user moves to another room, say the living room, the interface must follow the user, adapting itself to the new location. The goal is to be as close as possible to the user in order to provide not only for the best sound/speech capture from the user and listening conditions when AISH is speaking to the user, but also to have location context in order to better interpret the needs of the user.

The second requirement highlights that independently of the type of information such as direction, priority or sensitivity, the interface must support all the interactions between the smart home and its users. For instance, the interface must be suitable to deliver alarms, receive command orders, answer user questions and interrogate the user if something detected or inferred by the AISH system as suspicious (e.g., no human sounds are captured by AISH during a long period).

Finally, the third requirement mandates that the seamless interface solution must follow the user if s/he goes out. This feature is deemed as very useful, since it allows the user to continue to interact with the smart home even when s/he is outside. As the user context outside the home is not as controlled and accurate as when the user is inside the home, this use-case constitutes a new requirement for the whole AISH project, effectively expanding the smart home to the places where the user goes. This does not mean the implementation of hyperspaces [49] (at least in this version) but it does require the smart home to delegate a lighter smart system to follow the user and to continue to provide for her/his safety, security, comfort and to help out the user, even when s/he is outside. To comply with this last requirement, we embedded the speech interface inside a mobile agent and the MA approach can now fully accomplish the three main requirements described above. Indeed, by resorting to a mobile agent platform, we are able to launch a mobile agent to follow the user inside the home (location aware). Additionally, by embedding speech synthesizing and speech recognition in the mobile agent, we ensure that alarms, answers to user questions and reactions to user commands can also be performed outside the smart home, since mobile agents have the capability to carry updated information, in this case a minimal snapshot of the main knowledge base. The third requirement is also accomplished through agents: the smart home infrastructure detects when the user goes out and reacts by launching/cloning an agent into the handheld device of the user. When the user returns home, the mobile agent returns to the home mobile agent infrastructure and updates the main knowledge base if new relevant facts were collected while the user was outside.

### 3.3. Specification

Virtual Butler is the AISH module dedicated to human-system interface. It relies in speech recognition and voice synthesis, resorting to mobile agents. Additionally, as AISH targets elderly individuals living alone, a high level goal is to explore the voice and speech capabilities to have the interface behave like a virtual friend to humanize the smart home system, reinforcing the fact that the whole system exists to serve the human user.

**Figure 2.**
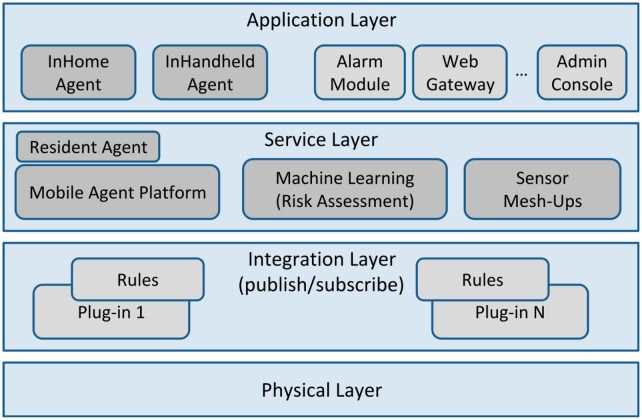
Integration of Virtual Butler in the AISH architecture.

Virtual Butler is comprised of: (i) mobile agents that provide its mobility and of (ii) an avatar that represents the face of the butler. The avatar has the capability of displaying expressions (smiles and sadness) and appears in the screens that are spread over the house. Figure 2 depicts the Virtual Butler subsystem when integrated into the AISH system architecture.

**Figure 3.**
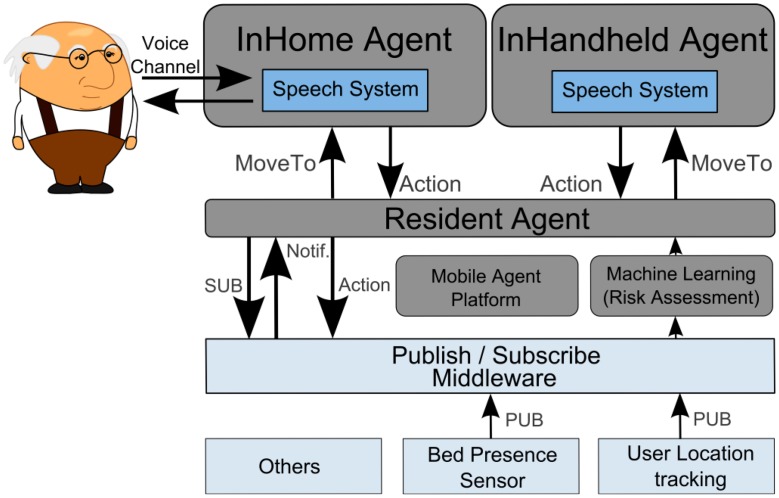
The Virtual Butler sub system.

The Virtual Butler subsystem is composed of three agents and the related mobile agent platform. The InHome Agent is used to “live” inside home and is sensitive to the user in-home location, while the InHandheld Agent is used to follow user when s/he goes outside. This agent is a subset of the InHome Agent so that it can be run on mobile platforms. The third agent, called Resident Agent, is a static agent that supports the other agents. Figure 3, presented next, details the Virtual Butler subsystem.

As can be seen in Figure 3, Virtual Butler is represented by the InHome Agent for interactions when the user is inside the home and by the InHandheld Agent when the user is outside the home area. As these two modules are mobile, there is a Resident Agent which bridges between mobile agents (no matter where they are located) and the smart home infrastructure. To be functional, the Resident Agent is notified by the Machine Learning module when an abnormal situation is inferred by this module. Apart from abnormal situations, the Resident Agent also subscribes channels (events) from the Publish/Subscribe middleware in order to be aware of the current smart home state. We argue that Publish/Subscribe based integration middleware is the best choice because it maintains the full decoupling among interacting modules, hence, reducing dependencies between them and easing updating and managing of modules. User location is one of the channels that Resident Agent subscribes in order to notify InHome Agent and InHandheld Agents to move near the user, depending on whether the user is inside or outside home.

Once InHome and InHandheld are fed with abnormal situations (if any) and with home state, they are ready to interact with the user through voice. The communication is two-fold. On the one hand, Virtual Butler should be able to answer a limited set of questions orally addressed to it. On the other hand, it should not only pass information, warnings and alerts on to the elderly, but it should also be able to trigger interactions (e.g., start a conversation) with its human counterpart when it senses an abnormality such a prolonged absence of sounds during daytime. For instance, it can ask if everything is OK and take action (contacting next of kin, emergency services, *etc.*) if the human fails to answer. Hence, the Virtual Butler (or the mobile agents) needs support for microphones and sound output from the hosting computer platform.

**Figure 4.**
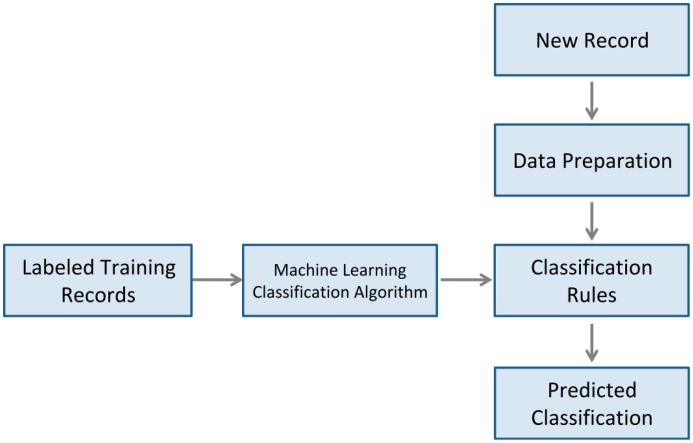
The stages of the machine learning module.

The Machine Learning module belongs to the first version of the AISH project and it was developed after AISH evaluation for mining the collected data. However, as it plays an important role in the Virtual Butler context, the module is briefly described next, with Figure 4 presenting the conceptual view of the machine learning module. The main aim of this module is to classify (inductive inference) the smart home states into two groups: normal situations or abnormal situations. In the future, this module is to be extended to also provide prediction in order to anticipate user actions. After the evaluation of the first version of AISH project which took three months, a conceptual prototype of a Machine Learning module was developed in order to gain knowledge from the recorded data (events). Data records include time periods, bed presence, in-house location, outside chairs presence, TV state, smoke and soil composites values. As during the first month we were calibrating and tuning the system, we have discarded events recorded in that period of time. Hence, only the events recorded in the two last months were used by the machine learning module, yielding about 1440 data records agglomerated by hour. The dataset was classified by us. Although being a conceptual prototype of a Machine Learning module, we have experimented with some classification algorithms resorting finally to the Weka framework (http://www.cs.waikato.ac.nz/ml/weka/). The algorithm which yielded the best results was the neuronal network algorithm Multilayer Perceptron, with the default configuration parameters. The dataset was validated using the cross-validation method using 10 folds and a split percentage of 66%. A good classification was achieved in 82.1% of the instances, with the remaining 17.9% being erroneously classified. However, as we are handling some critical alarm situations (e.g., presence of smoke), we do have to improve the precision of this method and we need to bias the system to, whenever in doubt, classify an occurrence as an alarm situation. Our research group considered those results very promising (for a conceptual prototype of a Machine Learning module) and currently there is a team focused into enhancing the Machine Learning module.

In order to be aware of the smart home state, the Machine Learning module subscribes all the channels used in the training data from the publish/subscribe middleware. Every time a new event arrives to the Machine Learning module, classification rules (e.g., trained algorithms) are applied to the last home state as a whole. Hence, the Machine Learning module implements a buffer that stores the last home state and is updated every time a new event arrives. If the classification stage predicts an “abnormal situation”, the Residential Agent notifies Virtual Butler which then takes action, like, for instance, asking the user if everything is OK.

## 4. Prototype

**Figure 5.**
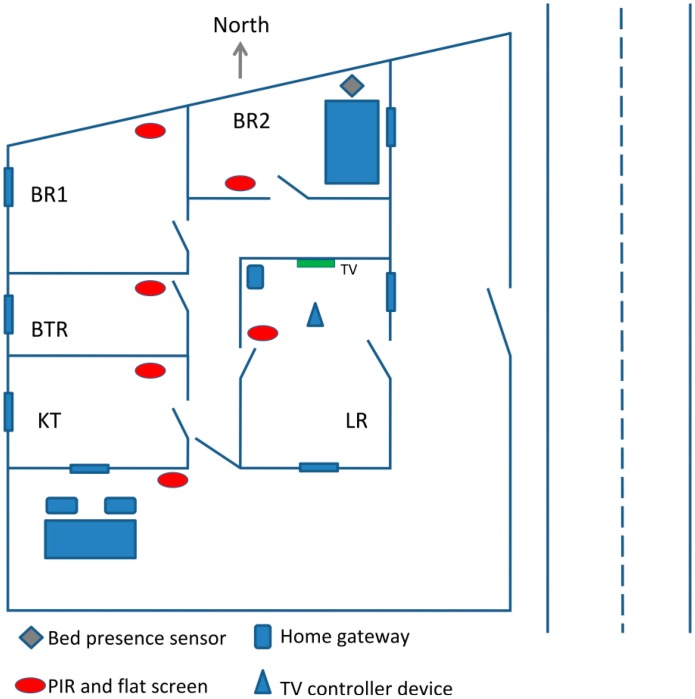
The home (LR-living room, BR-bedroom, BTR-bathroom, KT-kitchen).

The evaluation of the architecture described in the previous section was carried out in a real elderly person's smart home, where other ongoing projects of our research team were also evaluated and/or are being tested [17–19]. The volunteer's home is situated in a small rural village. The house comprises a kitchen, a living room, a bathroom, and two bedrooms. Around the house, there is a fenced open space with an outdoor table and two chairs. To make the home AIST-like, we installed some sensors, namely temperature, smoke, soil composite, humidity, presence, light and bed presence sensors. The house was also fitted with actuators, namely door lockers, portable AC controller, a customized IR remote to control TV and some RF light switch bulbs. Figure 5 shows the home sketch with relevant sensors. The home includes also temperature, smoke and humidity sensors in each room, and soil composite sensors in both external and internal potted plants. Furthermore, a light sensor is coupled to a presence detector to turn on the external light when needed. The home also features the fall detection prototype that has been developed in the context of another research project of our team [18–20].

### 4.1. Agent Platform

As stated earlier on, the implementation of the Virtual Butler relies on the mobile agent platform JADE [30–32]. Firstly, JADE implements a MA-based system where agents can be created, deleted, cloned and migrated. These operations can be made using Java API or through a native agent management GUI. Among other things, the GUI can be used to launch dummy agents, for example. This utility GUI agent can also be used to send Agent Communication Language (ACL) messages to other agents, receive and inspect messages from agents and read and save messages from/to file [32]. Secondly, since JADE is Java-based, it can be run over a variety of operating systems. Thirdly, JADE features a native agent communication system that can be used by the agents to negotiate among them. Fourthly, JADE is open source, actively maintained and includes reasonable documentation. Finally, JADE supports agent deployment in servers, personal computers, the Android platform and J2ME CDC and CLDC profiles.

The JADE platform is organized in containers and relies on native agents. As the name implies, the main platform container is an essential element of the system. It is within the main platform container that other containers need to register. These other containers can be located at other devices. Furthermore, the main container holds two native agents: (i) the Agent Management System (AMS) which provides naming service (uniqueness) and (ii) the Directory Facilitator (DF) that implements a Yellow Pages-like service useful to seek agents that implement a given set of services. In the perspective of the AISH project, the JADE platform main container resides in the AISH gateway computer. Mobile agents require a working network connection for moving themselves among target displays which arises problems in terms of communication with the base station. This challenge was overcome through the creation of a non-moveable agent which remains at the gateway, thus acting as a bridge between the moveable agents and the home infrastructure. There are two moveable agents: one covering the in-house and another one running on the Android mobile phone.

### 4.2. Resident Agent

**Figure 6.**
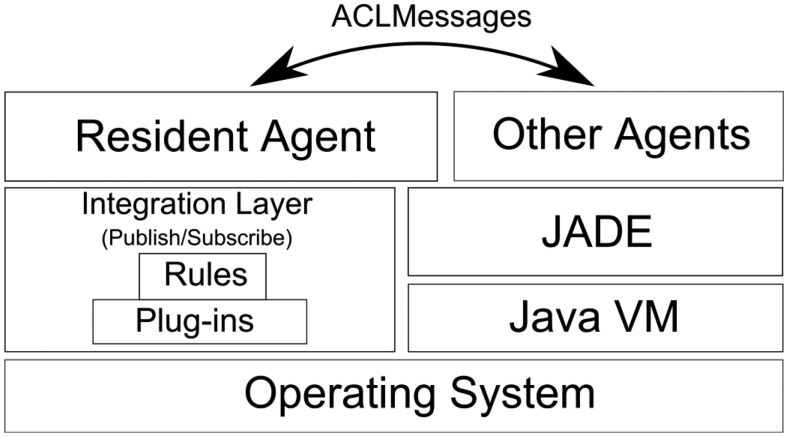
Resident and other JADE agents in the software stack.

The Resident Agent (RA) is a static Java JADE agent hosted on the AISH gateway computer. The main function of the RA is to be aware of the home state and to make its awareness-knowledge available to the other mobile agents. The RA gathers awareness data through both AISH publish-subscribe channels and machine learning components. The former follows the push paradigm over AISH publish-subscribe channels, namely the ALARMS and USER_LOCATION channels. The latter requires queries in order to get information about abnormal situations identified by the machine learning module. Within the network, the RA acts as base-station, making available its data through messages that it distributes to listening (other mobile) agents. In terms of the JADE stack, RA solely implements the network part of it, since it only needs to be able to pass data over listening agent through messages. Technically speaking, RA is a Java class which extends *jade.core.Agent* and uses ACL types to push data to other agents (Figure 6).

### 4.3. InHome Mobile Agent

The InHome Mobile Agent (IHMA) is a mobile Java JADE agent which follows the user when s/he is inside the house. IHMA implements three distinct technical features: (i) it listens to RA ACL messages (user location, alarms, warnings, *etc.*); (ii) it displays the avatar in the device it is running; (iii) it is voice-aware, both listening and producing speech whenever the need arises. The listening of messages is carried out through a JADE CyclicBehaviour and a message template to filter messages. Depending on the received messages, IHMA may need to inform the user, through voice synthesis, about alarms or other events or it may need to move into another device located in another room. For the purpose of mobility, there is a JADE container per room, all of them indexed by the AISH positioning system. For performance and independence purposes, each mobile agent keeps a local knowledge base, implemented through a local tuple space. This approach reduces the dependence on the Resident Agent, making the system more robust.

Regarding mobility, IHMA moves to the output device which is nearest to the user. For this purpose, each room includes a low cost flat screen connected to a low cost board computer such as a Raspberry PI. Whenever the IHMA needs to communicate with the user, the IHMA may activate its own avatar through the most appropriate flat screen device, that is, the device which is located in the room where the user is. Specifically, the avatar is activated in response to a message sent by the RA to the IHMA.

To show the animated avatar (GUI), the IHMA Java class extends the *jade.gui.GuiAgent* class. This is needed since the JADE agents are single threaded, resorting to a cooperative scheduling for task execution. In the current prototype, when IHMA speaks to the user, the avatar is updated through a simple animated gif mechanism, although no synchronization is yet implemented between the voice and the lips movement of the avatar. We aim to improve the interface as future work.

**Figure 7.**
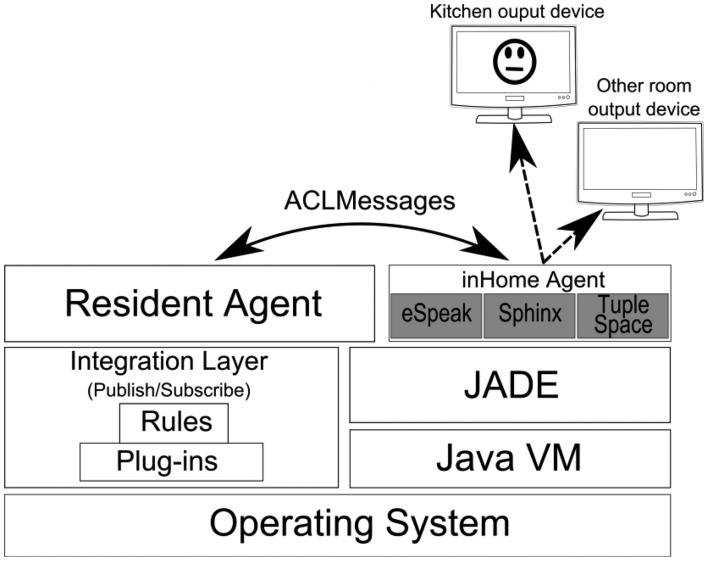
InHome Agent in the software stack.

In this voice-oriented AISH prototype, voice support is implemented through open-source and/or free speech engines. For speech synthesizing, we resort to the well proven eSpeak [50]. Regarding speech recognition, we tested two solutions: (i) one based on the CMU Sphinx-Speech Recognition Toolkit [37] and another one (ii) based on an Android smartphone. For the Sphinx-based solution, we resorted to a neck-hanging wireless microphone and installed the proper support in all JADE container devices. In the Android-based solution, audio is captured by the smartphone and converted to text. Then, the local agent that runs on the Smartphone sends the text to the IHMA agent, which processes it. To improve the audio capture, the Android device is relatively small so that it can be used in a neck-hanging position with the microphone facing the upper chest of the user, in the proximity of her/his mouth. The selection of the active speech recognition system (either offline Sphinx or online Android) is achieved by sending an ACLMessage to the RA, which then notifies IHMA to appropriately activate the selected method and disengage the other one. The whole process described in this section is summarized in Figure 7.

### 4.4. InHandheld Mobile Agent

**Figure 8.**
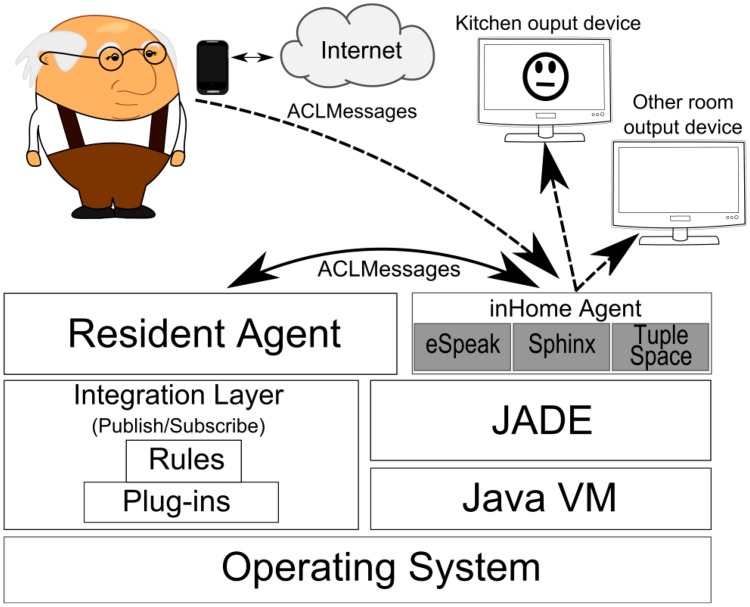
InHandheld Agent in the software stack.

The InHandheld Mobile Agent (IHAMA) represented in Figure 8 (as a smartphone) is a Java JADE-Android agent that runs in the Android smartphone which is carried by the user. This agent is static since the JADE-Android has no support for mobility. The IHAMA can play two distinct roles, depending on the location of the user: (i) it can be used for speech recognition for IHMA and (ii) it provides the functionalities of the Virtual Butler whenever the monitored user is outside home. Specifically, when the user is inside the home, the IHAMA only awaits the text produced by the speech recognition layer and forwards it to the IHMA to act upon. On the contrary, when the user is outside home, IHAMA effectively acts as the Virtual Butler, verbally passing alerts and other information to the human user.

**Figure 9.**
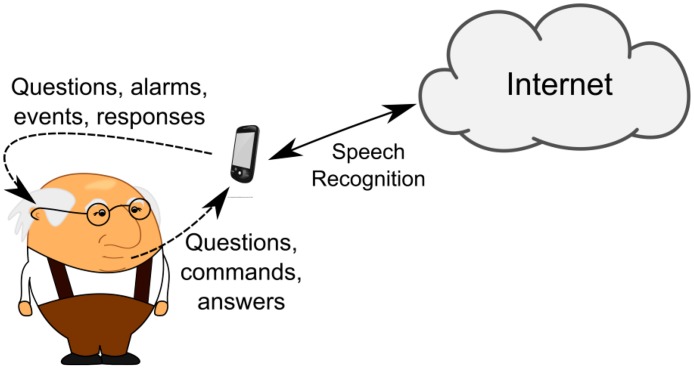
InHandheld Agent in the Android smartphone.

Like IHMA, the IHAMA relies on a tuple space-like structure to store a snapshot of the smart home infrastructure knowledge base. Hence, this agent can also perform checks such as asking the user if everything is alright when it detects, through its internal sensors, that the user has not moved for a time period larger than a predefined detection threshold. If the reaction provided by the user indicates that s/he needs help (for instance, the user fails to answer the question or s/he explicitly asks for help), the Virtual Butler acts upon by going into emergency procedures. At the same time, Virtual Butler also records the event for future update of the home knowledge database. We pursue this use-case in the evaluation scenario covered in the next section. Figure 9 shows the interaction between InHandheld Mobile Agent (smartphone) and the user.

## 5. Evaluation

As previously noted in the last section, the evaluation of the AISH prototype, as well as some other developments from complementary projects took place in a real household of a single elderly person. The volunteer inhabitant is an 82 year old female who has some mobility constraints. She rests at home for the main part of the day, besides taking care of the house. Additionally, as a hobby, the volunteer nurtures flowers kept outside the house and maintains in-house potted plants. She has no pets. Her routine is complemented by frequent small trips to the open area that surrounds the house, where the person can enjoy the natural light and the fresh air of the rural village, as well as engaging in small talk with neighbors and bystanders.

Regarding AISH, the house features a custom smart mailbox, a carryover from the first prototype. Indeed, the first AISH prototype was evaluated at the house for three months, yielding interesting and satisfactory results also submitted for publication. The backbone of the AISH infrastructure was used as a testbed for the Virtual Butler interface as presented in this work. The tests of the Virtual Butler lasted for three weeks, with *in-situ* visits from our research team occurring every three days.

We now describe the AISH backbone infrastructure installed at the testbed house, focusing on the specific sensors and actuators installed for the evaluation of the first prototype and which were also used for the Virtual Butler *in-situ* assessment. Figure 5 shows the locations of the AISH components throughout the house.

Besides the gateway computer which sits at the living-room, and as stated earlier, each room of the house has temperature, smoke, humidity sensors and also a Raspberry Pi class computer connected to a low cost small flat screen. All computers are connected to the WIFI network. Additionally, each low cost computer includes an USB PIR motion detector, which has detection capability up to a range of around six meters. The location detection inside the house is performed through the PIR motion detectors. An additional PIR is located outside the house, close to the main entry (this PIR is connected to the low cost computer that sits in the kitchen). Note that the location of the low cost computers was experimentally studied in order to achieve good reliability in movement detection. The data regarding movements (ON/OFF) captured by the PIRs are then published by the low cost computers to the home gateway computer through the WIFI network.

To allow the main computer to interact with the TV, we installed a special module which can control the TV set, namely control the volume, change channel or switch the TV ON/OFF. A use-case for this functionality is to automatically reduce the TV volume when the AISH needs to transmit an oral alert. Technically, the module interacts with a current detection sensor, in order to increase reliability when turning the TV ON/OFF. The custom-made TV control communicates with the home gateway through an RFM12b RF channel.

To detect the presence in bed, we resort to a Sharp distance sensor connected to a Mica2 sensor node. A similar installation is also used for the two outside chairs. The AISH also comprises other Mica2Dot and Mica2 with MTS300CA Sensor Board, as well as some physiological sensors, but they are less relevant for the evaluation of the Virtual Butler prototype.

After the evaluation of the first prototype, and as reported earlier on, we have trained a neuronal network (Machine Learning module) using the following data/sensor channels: (i) time period, (ii) bed absence time, (iii) in-house location, (iv) outside chairs presence, (v) TV state, (vi) presence of smoke and (vii) soil composite, in order to classify home state as normal situation or abnormal situation, every time the home state changes, even for just one sensor channel (see Table 1).

According to the possible values sensor readings could return, we have classified the training records as normal or abnormal home state in order to generate rules for training the neural network (multilayer perceptron). After the training phase, the inferred classification rules were used to classify the home state for each new sensor reading. As already reported in Section 3.3, the data set was validated using the cross-validation method using 10 folds and a split percentage of 66%. A good classification was achieved 82.1% of the instances, with 17.9% yielding an erroneously classification.

### 5.1. Voice Commands

The tested configuration of the Virtual Butler recognizes the following (in Portuguese):

- What time is it?
- Turn TV off.
- Turn TV on.
- Turn off bedroom light.
- Turn on bedroom light.

### 5.2. Alarms and Events

Alarms and events are grabbed by the Resident Agent by subscribing sensor channels in the publish/subscribe middleware (events) or by listening to the output of the machine learning classification algorithm (alarms).These events are conveyed by the Virtual Butler through voice synthesizing. For example, the Virtual Butler orally reports “new mail” (in Portuguese) when the instrumented mailbox detects a load event. On the other hand, by subscribing the smart mailbox channel, the Resident Agent knows if there is or not mail in the box and reports that event to the user. Through the output of the machine learning classification algorithm, the Resident Agent can identify an abnormal situation and act upon it. The same alarm is handled in periods of 3 min (for instance, the presence of smoke), while the same existing event has a period of 30 min (for instance, existence of mail in the mail box). The period of time for handling the same recurring alarms and events is easy configured in the system (Resident Agent).

### 5.3. Abnormal Situations

We define abnormal situations as special situations that might indicate that something is out of the ordinary, and thus might require the immediate intervention of Virtual Butler, unlike events which are less urgent. For our experimental evaluation, we considered the following abnormal situations:

- The user gets up at late night and is out of the bed for a period larger than usual (threshold = 30 min).
- The user is at bed longer than usual.
- The user is outside home at nighttime.
- There is smoke inside the house.
- Potted plants are thirsty.

Whenever one of these situations is detected, the Virtual Butler orally asks the user if everything is fine and reacts accordingly to the user's (or lack of) answer. The expected answer is (in Portuguese) “It is ok!”.

### 5.4. Evaluation of the Virtual Butler Movements

**Figure 10.**
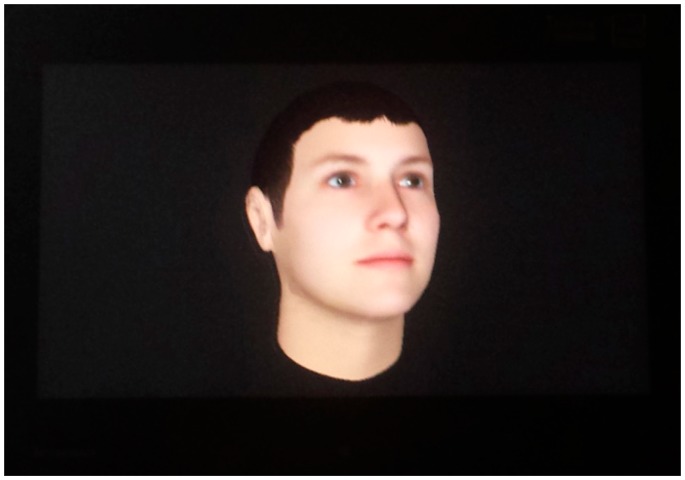
The Virtual Butler avatar.

The assessment of Virtual Butler (avatar in Figure 10) focused on two different perspectives: (i) technical behavior and (ii) user analysis. The technical behavior assessment involves comparing the logs of the smart home infrastructure with the ones of Virtual Butler, verifying the actions of Virtual Butler in response to events or alarms detected by the smart home infrastructure. For example, if the log of the smart home infrastructure reports “user enters kitchen” at 11 AM, then the log of the Virtual Butler should have an 11 AM entry “moved to kitchen”, otherwise something went wrong.

Our cross analysis between the logs of the smart home infrastructure and of the Virtual Butler showed that it reacted as expected for all the logged events. Measurements of the reaction time, that is, the time interval that mediates between the reception of a “move to room” event from the Resident Agent and the correspondent availability of the mobile agent in the new room is, on average, 2.2 s. We call this time interval the reaction time to movement (RTM) of Virtual Butler. The minimum, average and maximum values for the RTM are 1.7, 2.2 and 4.2 s, respectively. We believe that an RTM of 2.2 s, which means that the Virtual Butler has a 2.2-second lag when the user enters another room, is acceptable considering a human time scale.

### 5.5. Evaluation of Response to Voice Commands/Questions

The responses of the Virtual Butler to voice commands/questions were assessed considering two different input devices: (i) a portable wireless microphone and (ii) a small size neck-hanging Android smartphone. As stated before, the Android smartphone can behave as an agent host or as a portable microphone. This behavior is defined by the InHandheld Mobile Agent according to the user location, that is, whether the user is inside or outside home.

#### 5.5.1. Wireless Microphone and CMU Sphinx Speech Recognition

The wireless microphone used is a typical portable neck-hanging wireless microphone. The wireless microphone together with the CMU Sphinx software stack (set with the appropriate domain specific language model) implements the speech recognition feature. It is used regardless of whether the user is standing or moving around the house.

While the neck-hanging wireless microphone yielded interesting results in a laboratory setup, the recognition ratio achieved at the real smart home were rather disappointing, with solely the *yes* and *no* words reaching a satisfactory recognition ratio. We believed that the recognition ratio can be improved by using a more appropriate data training set for the Sphinx software, since elderly voices are substantially different from younger peoples' voices [2]. We plan to address this issue in future work.

Another major hurdle for the wireless microphone was the fact that the elderly testing user only used it for short periods after the regular team visits. Indeed, the user never took the initiative of wearing the microphone and never recharged the batteries (the microphone was left operational after each of our visits). In fact, the wireless microphone was considered by the volunteer user as something strange to live with.

#### 5.5.2. Android-Based Speech Recognition

Inside the house, the Android smartphone is used for voice capture and speech recognition. Even with some background noise, the smartphone-based solution achieved a high recognition ratio for both words and sentences. Moreover, the software required no training stage or preparation.

Each time the Android agent recognizes a word/sentence, it sends the corresponding text to the Resident Agent. The RA, besides logging the text, passes the word/sentence to the smart home infrastructure which then performs the corresponding action or delivers the appropriate answer. When an oral answer is needed, the synthesized voice goes through the speakers of the interface which is displaying the Virtual Butler.

For the evaluation of the Android speech recognition ratio, we resorted to log analysis. Overall, only 6% of speech could not be recognized by the Android recognition software, meaning that 94% of words/sentences were correctly identified. Considering the existence of noise, although moderate, proper of a small rural environment, this high ratio is quite an achievement. Regarding the identified commands, the results of the corresponding reactions are presented in Table 2.

The results show that the answer for the question “what time is it?” was always successful. The TV ON/OFF commands achieved, on average, 75% success. This means that 25% of the commands were successfully detected, but that the corresponding commands did not actuate properly on the TV set. We hypothesize that the 25% failures are due to the lack of accuracy of the TV control device. Indeed, although the device always sends the proper command, the TV fails to react accordingly. Note that the controlling TV device repeats a non-executed ON/OFF command up to three times. Detecting whether the ON/OFF command was properly carried out by the TV is performed by the sensor that measures the electrical power consumed by the TV set. Finally, the “Turn OFF/ON bedroom light” commands were not assessed since the user never used them.

Unfortunately, and similarly to what happened to the wireless microphone, the smartphone was only used during and for short periods following the visits of the research team. The user pointed out that she was not comfortable to carry what she considered an expensive device for which she had no knowledge to operate properly. Even the simple operation of recharging the phone battery was deemed too complicated and left to our research team (to solve the battery issue, we used a spare battery, swapping it into the smartphone at the beginning of each visit of the research team).

Overall and due to the low usage of the sound capturing devices (wireless microphone and smartphone) the presented results and statistics need to be taken with great care, since the assembled data set is small. An important lesson from the (lack of) interaction of the user with the input sound devices, is that an elderly person who had previously never directly used a mobile phone (let alone a smartphone), do not feel motivated to use a strange neck-hanging device all the time. The same feeling occurred with the smartphone, deemed too fragile. Nonetheless, and despite the difficulties in having the sound capture device accepted by the elderly, we consider the audio interaction a must have for AAL that targets elderly individual living alone.

### 5.6. Evaluation of Abnormal Situations Handling

An abnormal situation requires special treatment from the smart home infrastructure, and correspondingly, requires special handling by the user/smart home infrastructure interface. For instance, whenever an abnormal situation is detected, the TV volume is muted to avoid sounds coming from the TV to interfere with the smart home microphones and speakers.

**Figure 11.**
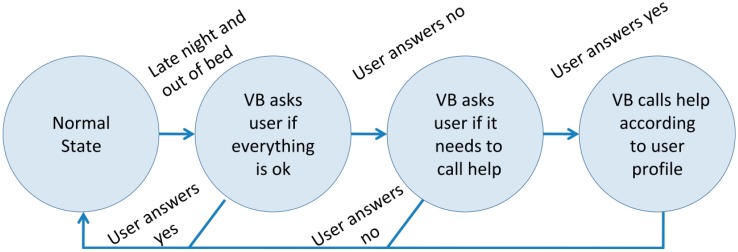
Algorithm used by Virtual Butler to handle abnormal situations.

The AISH project handles abnormal situations using a simple algorithm (see Section 5.4 for the list of abnormal situations handled in this prototype). Next, we present the algorithm to handle the situation in which the user gets up late night and spends more time than considered usual (currently set to 30 min) in the restroom or in another home room (see Figure 11). In the tested prototype, we apply the same generic algorithm to handle all situations that are flagged as abnormal.

During the assessment of the prototype, many situations were flagged as abnormal, most of them related with time out of bed at late nighttime. This was caused by the initial rather short threshold tolerance interval (15 min) that triggers the alarm mode response (then changed to 30 min). This interval did not effectively match the user routine, which varied quite a lot. This is understandable for someone who rarely has a fixed schedule. Since the user rarely used the speech capture devices, all abnormal situations went unanswered and thus escalated to emergency mode. For the prototype assessment, the emergency mode simply logged the occurrence and alerted the research team. If deemed appropriate, a phone call was made by our research team to assess the seriousness of the event.

### 5.7. Evaluation of the Android Agent

As stated earlier, the main goal of the Android agent is to have the possibility of following the user outside of the house. However, as previously reported, a smartphone is not considered an everyday object for our volunteer and thus opportunities to test it under a real scenario were slim. In fact, the elderly lady only used the Android smartphone during the visits of the research team who encouraged her to use it outside home, namely around the table located outside. We then fired abnormal situations *on demand* like the “potted plants are thirsty” situation and recorded the behavior of the user and of the AISH system. Often, we had to stimulate the user to provide an answer to Virtual Butler when asked if everything was alright. This is yet another proof that a smartphone is not a friendly device for older people that have never used or had one. However, it is important to point out that the elderly individual enjoys relatively good health considering her age and has not, to the best of our knowledge, suffered any major health scare. We believe that elderly less healthy might be more willing to accommodate into their daily routines devices that might assist them if the need arises.

### 5.8. Volunteer Interview

We interviewed the volunteer to collect her opinion about the AISH prototype, with emphasis on Virtual Butler. Additionally, we sought to compare the results obtained from the log analysis with the opinion of the volunteer. The interview relied in the following four major questions:

1. What do you think about the Virtual Butler that follows you inside home?
  *Answer*: *It is ugly and does not seem happy*. *However it is a good idea having a virtual friend that talks to me*.
  A new and more appealing avatar is being built with special focus on synchronization of the mouth animation with the speech duration. However, it is relevant to note that the user liked the idea of a Virtual Butler (assistant) and effort must be employed in order to enhance it both graphically and to increase its knowledge and dialog capabilities.
2. What do you think about the neck-hanged devices (microphone and smartphone)?
  *Answer*: *I do not like them at all! I really don't want to use them especially around the neck, as they are uncomfortable. Besides, they need to be recharged, something I tend to forget*.
  We agree with this point of view. However, since the output interface has to deal with special events or situations (critical alarms), the AISH system needs, with a very high level degree of certainty, to capture the user answers if one is provided and, as importantly, to properly detect when none is provided in a timely manner. A possibility that we consider for future work is to replace the tested microphone devices by smaller ones, possibly hidden from outside view within a necklace or a ring.
3. What do you think that first prototype accomplish better than this second prototype?
  *Answer*: *the automation of lights and TV is better than having a gadget to control appliances by voice. There is no need to have these voice commands when the house already learned when to turn on/off TV and lights*.
  We agree with this point of view, but the idea was not to turn off and on lights and TV for the future but just test the sample voice commands in order to assess the quality of speech recognition with simple operations.
4. Do you have any complains about the prototype that you would like to share with us?
  *Answer*: *In fact I have. When the Virtual Butler starts speaking by its own initiative, it sometimes scares me*.
  We believe that this can easily be fixed, by using an enjoyable low intrusive sound just before the Virtual Butler speaks (for instance, similar to the chime heard in shopping malls and airport before a speaking announcement).

## 6. Conclusion and Future Work

We describe and evaluate a mobile agent-based Virtual Butler for smart homes which main novelty is to interact with the inhabitants of the house through a voice interface. Specifically, the Virtual Butler aims to fulfill two main requirements: (i) be location-aware, behaving accordingly to the user location in the AISH and (ii) be able to fulfill all the human-machine interaction of the AISH setup through computer-generated speech and voice recognition.

A working prototype of the Virtual Butler was tested and assessed in a live environment, more precisely in an AISH-adapted house of an 82 years old volunteer lady. Based on this assessment and on the feedback gathered through an interview with the volunteer, we conclude that the location-aware module of the Virtual Butler is working as specified, thus fulfilling the first requirement. Regarding the human-interaction interface, no definitive conclusion can be drawn, since the volunteer barely interacted with the Virtual Butler. However, two valuable lessons were learnt: firstly, wireless microphones and neck-hanged smartphone are regarded as strange by people that never used this kind of devices. Moreover, they are not comfortable. Secondly, several issues impaired the functionality of the seamless voice-based interface. In particular, resorting to battery-operated devices with a rather low capacity (few days at the most) hinders the operability of the system, especially when dealing with technology-impaired users. Another issue, although easily fixable, arises from having a computerized voice intervening without prior notice, a situation that can stress the user.

Based on the conclusions and learnt lessons, we are already specifying future work that can, hopefully, better suit the Virtual Butler to the needs and demands of smart home, and more importantly, of their inhabitants. Firstly, we are currently researching alternatives to the used wireless microphones that can simultaneously have better acceptance by the users, has no dependency over batteries and require few or no maintenance at all. Ceiling microphones are a possibility that solve both the reported discomfort of carrying a microphone and the battery issue. However, as reported by Vacher *et al.* [2], using ceiling microphones significantly impacts the quality of captured sound, not only because of the distance between the user and the microphone, but also because the user might not be facing the microphone. Additionally, ceiling microphones are prone to capture unwanted environmental noises. Thus, we are still open of having the user wearing a device that can interact with system. For this purpose, we plan to research the possibility of developing a tiny device with great battery autonomy, similar to a panic button device, but fitted with two simple YES and NO buttons. This device would also act as a complement to the ceiling sound capture, used whenever the situation requires a high level of confidence over the user input or, as importantly, lack of answer.

An identified limitation of the AISH infrastructure that supports the Virtual Butler is the staticity of the Android agent. Indeed, the JADE underlying middleware does not support mobility in the Android platform. This forces the Android agent to rely on inter-agent communication, resembling more a client/server approach than a true mobile agent paradigm. Another limitation of JADE is that it does not support other mobile platforms, namely Apple iOS and Windows Phone. We plan to address these issues in future work.

To reduce costs both at the hardware level and at the power consumption level, we plan to replace the per room low cost computers by fewer ones, each one possibly feeding several output displays. This approach should also reduce maintenance issues since there are fewer computers. Another item on our future work list is to enrich the Sphinx domain specific language model to cope with variants of base expressions. Another improvement is to enlarge the detection of TV OFF in expressions like “Turn TV OFF”, “TV OFF”, “Shutdown TV” and “OFF TV”. We also plan to address the glitches regarding the TV control device, that is, the multiple consecutive malfunctions that occurred on the live AISH experiment with the “shut down” and “power on” commands. Lastly but not least, the assessment of the next version of the system needs to involve multiple volunteers in order to attain a wider representation.

## Figures and Tables

**Table 1.** The sensor channels training set.

**Sensor Channel**	**Data Type**
Time period	Early morning, morning, …
Not in bed portion of time	0, 1, 2, … portions
In-house location	The room where user is
Outside chairs presence	0 / 1
TV State	0 / 1
Smoke	0 / 1
Soil composite	0..1023

**Table 2.** Results for voice commands using Android speech recognition.

**Action**	**Recognition Ratio**
What time is it?	100%
Turn TV off	78%
Turn TV on	72%
Turn off bedroom light	---
Turn on bedroom light	---
